# Anti-Ri-Associated Paraneoplastic Neurological Syndrome Revealing Breast Cancer: A Case Report

**DOI:** 10.7759/cureus.21106

**Published:** 2022-01-11

**Authors:** Rim Tazi, Zakaria Salimi, Hajar Fadili, Jehanne Aasfara, Asmaa Hazim

**Affiliations:** 1 Neurology, Cheikh Khalifa Ibn Zayed Hospital, Faculty of Medicine, Mohamed VI University of Health Sciences (UM6SS), Casablanca, MAR

**Keywords:** paraneoplastic neurologic syndrome, opsoclonus-myoclonus, cerebellar syndrome, anti-ri antibodies, metastatic mammary adenocarcinoma

## Abstract

Neurological paraneoplastic syndromes are a rare entity that affects patients with cancer. Anti-Ri antibodies affect the brain stem and produce a heterogeneous rapidly progressive subacute syndrome depending on the involvement of the different regions concerned. The most common clinical presentation is opsoclonus-myoclonus syndrome and paraneoplastic cerebellar degeneration. Here we report a case of a 60-year-old woman with a subacute static-kinetic cerebellar syndrome, cervical dystonia, and multiple cranial nerve palsies revealing a mammary adenocarcinoma. Anti-Ri antibodies were positive in her blood. Our observation underscored the importance of the identification of the tumor for early treatment management to avoid irreversible neurological manifestations.

## Introduction

Neurological paraneoplastic syndrome is a rare entity that affects patients with cancer. We report the case of a female patient with cerebellar syndrome and multiple lesions in cranial nerves revealing breast cancer. Anti-Ri antibodies were positive in her blood. In spite of normal MRI brain findings, the study of cerebrospinal fluid and the search for onconeuronal antibodies are important to label the paraneoplastic origin of neurological symptoms. The identification of the underlying tumor is essential for early treatment management to avoid irreversible neurological damage.

## Case presentation

**Figure 1:**
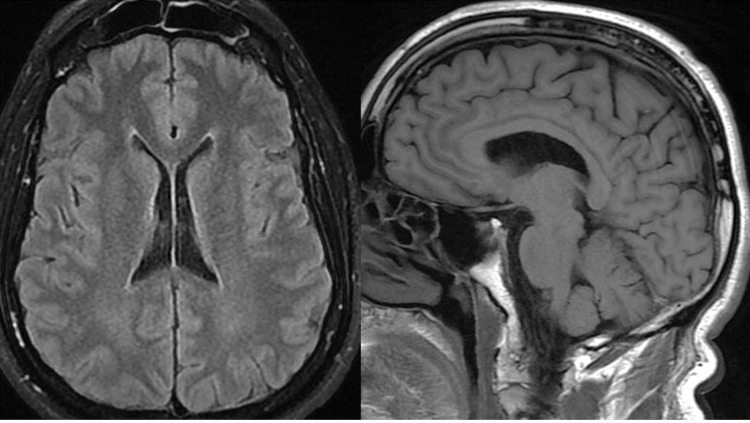
Brain MRI resonance with contrast shows no cerebral abnormality.

We report a case of a 60-year-old woman with a surgical history of right mammary lumpectomy done in 1983 without further chemo or radiotherapy. She presented four months ago with subacute dizziness with severe vomiting leading to a considerable loss of weight. Her family noticed impaired swallowing and explosive speech. Her clinical symptoms were also associated with binocular diplopia, eye deviation, right ptosis, left facial weakness, and involuntary contracture of neck muscles on the right side. The clinical examination on admission found a static-kinetic cerebellar syndrome and cervical dystonia. She had ptosis of the right eye with limited abduction, depression, and the pupil does not react to light. The left eye cannot move outward. We also noticed a facial decreased sensation of the left side. Rankin's score was 4. The brain magnetic resonance imaging with contrast showed no abnormalities (Figure 1). The cytological study of the spinal puncture was <5 leucocytes/μl without any visible suspicious cells.

The etiological assessment was found to be negative, including autoimmune diseases, viral serology, angiotensin-converting enzyme, and antiphospholipid syndrome. The thyroid assessment as well as the vitamin D and B12 levels were normal. After excluding all other potential causes of the neurological symptomatology, the most probable etiology left was of paraneoplastic origin. A search for onconeuronal antibodies has been launched; anti-Ri antibodies came back positive. The whole-body CT scan was negative. She was given 1 g per day methylprednisolone for five days. The patient started to improve clinically on day five with gradual reversal of her ptosis and diplopia.

**Figure 2:**
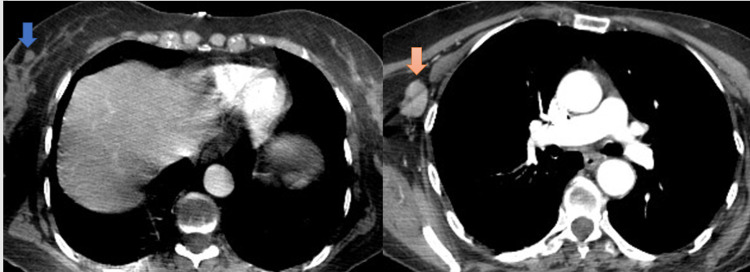
Chest CT scan with IV contrast shows right axillary lymphadenopathy IV: Intravenous; CT: Computer tomography Left-hand side figure panel: the blue arrow shows a small right breast nodule measuring 7.4 mm on the short axis Right-hand side figure panel: the peach-colored arrow shows right axillary lymphadenopathy measuring 25.7 mm on the long axis and 16.3 mm on the short axis

After three months, she presented a palpable lump in the right axilla. The CT chest with intravenous (IV) contrast revealed right axillary lymphadenopathy with cystic consistency. It measured 25.7 mm on the long axis and 16.3 mm on the short axis. The CT chest scan also showed a small right breast nodule localized on the upper and outer quadrant. The short-axis diameter was 7.4 mm (Figure 2). The breast ultrasound showed a small, irregular, hypoechoic right breast nodule and cystic right axillary lymphadenopathy.

**Figure 3:**
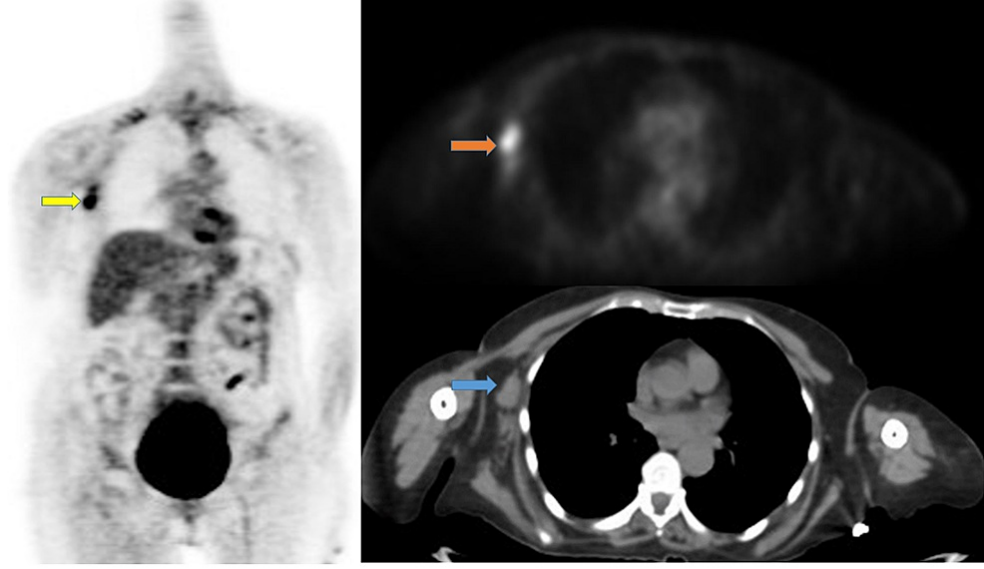
CT PET scan CT: Computer tomography; PET: Positron emission tomography Left-hand side figure panel: the yellow arrow shows hyper-metabolic right axillary adenopathy with a maximum standardized uptake value (SUVmax) of 7.9 Right-hand side figure panel: orange and blue arrows show hyper-metabolic right axillary adenopathy

The results of the positron emission tomography (PET) scan showed a hyper-metabolic right axillary adenopathy with two non-hyper-metabolic mammary nodules (Figure 3). Ultrasound-guided biopsy of the suspected adenopathy suggested breast invasive carcinoma with axillary lymph node metastasis. The estrogen receptor (ER) was positive at 90%. The expression of progesterone receptor (PR) was also positive at 80%. The immunohistochemical assessment of human epidermal growth factor receptor 2 (HER2) status was negative. The medical oncology team decided to begin hormonotherapy. The paraneoplastic neurological syndrome did not respond to this therapy. The patient's state after the hormonotherapy was the same. She had a static-kinetic cerebellar syndrome, cervical dystonia, and multiple cranial nerve palsies. Chemotherapy and surgery were planned. Unfortunately, our patient died from a septic shock. 

## Discussion

We reported the rare case of breast cancer revealed by neurological paraneoplastic syndrome with a strongly positive anti-Ri antineuronal antibody. Paraneoplastic neurological syndromes (PNS) were first described in 1968. It is a rare situation that affects 0.01% of cancer patients [1]. Paraneoplastic neurological syndromes (PNS) are associated with different tumor types such as thymoma, small cell lung cancer, breast cancer, ovarian cancer, lymphoma, and testicular cancer. Only 56 cases of paraneoplastic neurological syndromes related to breast cancer have been reported in the past 20 years [2].

Anti-Ri Antibodies affect the brain stem and produce a subacute paraneoplastic syndrome. The most common clinical presentations are opsoclonus-myoclonus syndrome and paraneoplastic cerebellar degeneration [3]. Our patient presented with a static-kinetic cerebellar syndrome, multiple cranial nerve palsy, and cervical dystonia. In this way, our results confirm the data found in the literature, the opsoclonus-myoclonus syndrome is not pathognomonic of anti-Ri antineuronal antibody [4]. 

**Figure 4:**
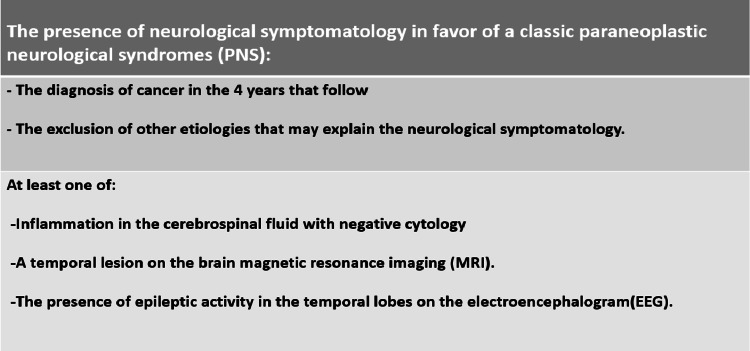
The diagnostic of paraneoplastic neurological syndromes.

The diagnosis of paraneoplastic neurological syndromes (PNS) is difficult to confirm due to the variability of symptoms and clinical pictures. In general, we should think about paraneoplastic syndromes (PNS ) in the event of any subacute, atypical, or multifocal neurological impairment in a patient over 50 years of age. The presence of anti-neuronal antibodies in the cerebrospinal fluid and/or in the blood is a sign of paraneoplastic syndromes (PNS). However, its absence does not rule out the diagnosis [5]. For this reason, a consensus of experts in neurology has defined criteria for making the diagnosis of paraneoplastic neurological syndromes (PNS) which are as follows (Figure 4) [6-7].

The pathophysiology of paraneoplastic neurological syndromes (PNS) is not fully understood. The most probable hypothesis is that breast cancer causes immunity cross-reacts with select neural tissues to produce a paraneoplastic syndrome [8]. Several studies also report that the alteration of the function of the *p53* gene is responsible for the expression of mutated proteins causing an autoimmune reaction or even paraneoplastic syndrome [8].

In the literature, a study proposes that an immunosuppressive treatment including, immunoglobulins (IVIg), cyclophosphamide (CTX), and methylprednisolone (MP), gives no-improvement in all patients who present paraneoplastic neurological syndromes (PNS) with Rankin score ≥4 [9]. It was not the case with our patient who received 1 g of methylprednisolone per day for five days. She started to improve clinically on day five with gradual reversal of her ptosis and diplopia but she still had the other neurological symptoms. In the end, the treatment of the primary tumor is crucial. Our patient has advanced ER-positive/PR-positive/HER2-negative breast cancer. She received hormone therapy with transient stabilization of her condition. In the literature, 75% of patients in a study revealed benefits from immunotherapy associated with their cancer treatment [2].

This article discusses a rare case report of paraneoplastic neurological syndrome. The specific paraneoplastic (anti-Ri antibody) is associated with breast cancer patients who developed paraneoplastic syndrome. Neurological manifestations in our patient were characterized by static-kinetic cerebellar syndrome, multiple cranial nerve palsy, and cervical dystonia, which preceded the finding of breast cancer by three months. The breast lesion was impalpable on the clinical examination and asymptomatic for its very small size. The present case illustrates that the presence of the anti-Ri antibody may help to identify patients with ataxia and multiple cranial nerve palsy and cervical dystonia, who often suffer from breast tumors. This research fits within the existing literature as the opsoclonus-myoclonus is not pathognomonic of anti-Ri antibody.

The brain MRI and the whole computed body scan did not show any abnormalities. Despite the positivity of anti-Ri antibodies, the appointment of PET is reported due to the COVID-19 pandemic. This area of weakness in our research process led to a delayed diagnosis for three months.

## Conclusions

We described the rare case of breast cancer revealed by neurological paraneoplastic syndrome with positive anti-Ri antibodies. The opsoclonus-myoclonus syndrome was absent. We emphasized the importance of a large clinical spectrum of anti-Ri antibodies. The diagnosis of paraneoplastic syndromes is complicated and time taking, as in the case of this patient. The treatment of the primary tumor may contribute to a good prognosis but may not recover the neurological symptoms. Even if the diagnosis was late, our case fits within the existing literature. It highlighted the benefit of immunotherapy treatment for a stabilized neurological condition.
